# Treg suppression of immunity within inflamed allogeneic grafts

**DOI:** 10.1172/jci.insight.160579

**Published:** 2022-08-22

**Authors:** Hehua Dai, Andressa Pena, Lynne Bauer, Amanda Williams, Simon C. Watkins, Geoffrey Camirand

**Affiliations:** 1 Thomas E. Starzl Transplantation Institute, Departments of Surgery and Immunology, and; 2 Center for Biologic Imaging, Department of Cell Biology and Physiology, University of Pittsburgh School of Medicine, Pittsburgh, Pennsylvania, USA.

**Keywords:** Immunology, Transplantation, Adaptive immunity, Th1 response, Tolerance

## Abstract

CD4^+^Foxp3^+^ regulatory T cells (Tregs) restrain inflammation and immunity. However, the mechanisms underlying Treg suppressor function in inflamed nonlymphoid tissues remain largely unexplored. Here, we restricted immune responses to nonlymphoid tissues and used intravital microscopy to visualize Treg suppression of rejection by effector T cells (Teffs) within inflamed allogeneic islet transplants. Despite their elevated motility, Tregs preferentially contacted antigen-presenting cells (APCs) over Teffs. Interestingly, Tregs specifically targeted APCs that were extensively and simultaneously contacted by Teffs. In turn, Tregs decreased MHC-II expression on APCs and hindered Teff function. Last, we demonstrate that Treg suppressive function within inflamed allografts required ectonucleotidase CD73 activity, which generated the antiinflammatory adenosine. Consequently, CD73^–/–^ Tregs exhibited fewer contacts with APCs within inflamed allografts compared with WT Tregs, but not in spleen. Overall, our findings demonstrate that Tregs suppress immunity within inflamed grafts through CD73 activity and suggest that Treg-APC direct contacts are central to this process.

## Introduction

CD4^+^Foxp3^+^ regulatory T cells (Tregs) are essential in controlling immunity and inflammation in both secondary lymphoid organs (SLOs) and nonlymphoid tissues (1). For example, Tregs have been shown to suppress immunity in skin and in tumors (2, 3). In line with this, in transplantation, Tregs have been suggested to provide protection from rejection within allogeneic grafts (4–6). In addition to their suppressor function, Tregs have been shown to play a role in maintaining homeostasis and promoting tissue repair in peripheral tissues such as visceral adipose tissue, gut mucosa, and skeletal muscle (7, 8). Thus, there is great interest in harnessing Treg functions for therapeutic purposes in multiple disease settings.

In SLOs, Tregs can suppress through a multitude of mechanisms (e.g., cytotoxic T lymphocyte–associated protein 4 [CTLA-4], IL-10, TGF-β, granzyme B, IL-35, adenosine generation, IL-2 deprivation, and inhibition of antigen-presenting cells [APCs]; refs. 9, 10), suggesting that these mechanisms of suppression vary according to the local environment. In support of this, Tregs were shown to be adaptable to ongoing effector T cell (Teff) responses through the expression of concomitant transcription factors (11–14). For example, during a Th1 effector response, which is mediated by the transcription factor T-box transcription factor TBX21 (T-bet), Tregs also differentiate into T-bet^+^ Th1 regulatory cells. This differentiation enables Tregs to migrate to target tissues of Teff responses by expressing appropriate chemokine receptors and ligands (11). However, the mechanisms underlying Treg suppression in inflamed tissues, specifically in allografts, remain largely unexplored.

To address this, we used a murine model where either graft rejection by Teff or Treg suppression of rejection can only occur within inflamed allogeneic islet transplants. We show that Tregs protected from graft rejection by impeding Teff function, without affecting Teff proliferation and accumulation within transplants. Using 2-photon intravital microscopy (IVM), we demonstrate that both Teffs and Tregs accumulated in APC-rich areas and that Tregs spent most of their time in contact with APCs. Interestingly, Tregs preferentially targeted APCs that were simultaneously being contacted by Teffs. In turn, this led to a reduction in MHC-II expression on APCs and specifically to the inhibition of a subset of Teffs that were extensively contacting both APCs and transplanted islets. Last, the ectonucleotidase CD73 was required for the Treg suppressive function within inflamed allografts. Overall, our data demonstrate that Tregs counteract inflammation through CD73 activity and suggest that APCs are central to Treg suppressive function within inflamed tissues, such as an allograft.

## Results

### Tregs suppress Teffs without prior activation in SLOs.

**Figure 1:**
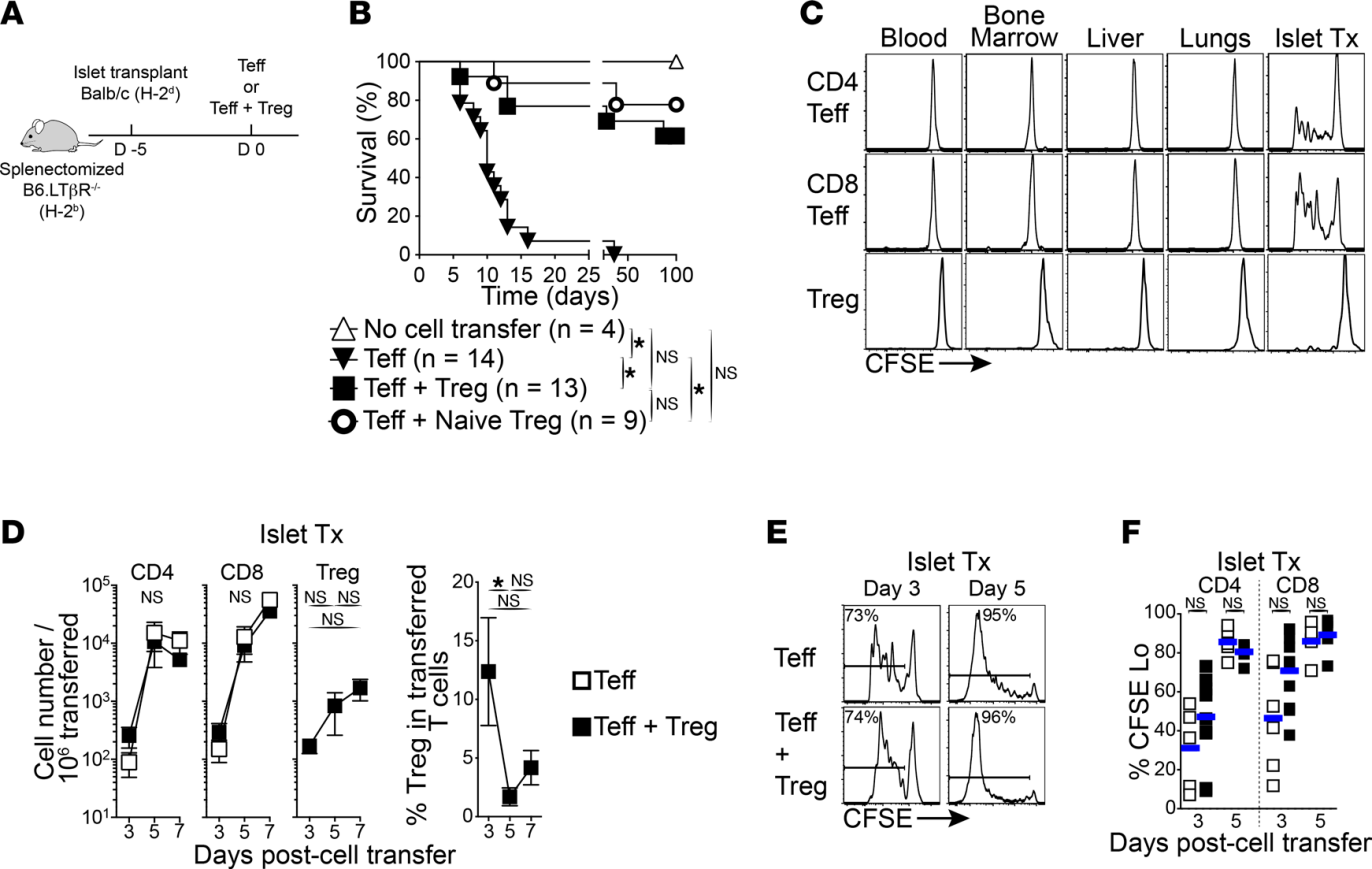
Tregs can prevent allograft rejection by Teffs in absence of SLOs. (**A**) Schematic diagram of experimental design: Streptozocin-induced diabetic mice lacking SLOs (splenectomized B6.LTβR^–/–^; H2^b^) were transplanted with islet allografts (Balb/c; H2^d^). After 5 days, the mice received 2 × 10^6^ to 3 × 10^6^ Teffs or Teffs + Tregs from congenic WT B6 or B6.Foxp3-RFP mice (H2^b^). (**B**) Islet allograft survival in mice lacking SLOs as described in **A** that received cell transfers as indicated. Tregs were from mice previously exposed to islet donor antigens (i.e., Balb/c), and naive Tregs were from naive mice. Log-rank Mantel-Cox tests used. (**C**) Graft-specific proliferation of Teffs and Tregs in absence of SLOs. Representative flow cytometry histograms of CFSE dilution in transferred Teffs and Tregs as in **A**. (**D**) Tregs accumulate in allografts over time but had no effect on migration and accumulation of Teffs. Left panels: absolute cell numbers of CD4^+^ and CD8^+^ Teffs, and of Tregs within allograft over time. Right panel: fraction of Tregs within the infiltrate of transferred T cells over time. Mean ± SEM. Multiple *t* tests with Holm-Šidák correction for multiple comparisons (Teffs alone vs. Teffs + Tregs at each time point; left 2 panels) and Kruskal-Wallis with Dunn’s multiple comparison tests (right 2 panels) were used. (**E**) Tregs do not affect Teff proliferation within allografts. Representative flow cytometry histograms of CFSE dilution in transferred CD8^+^ Teffs on days 3 and 5. (**F**) Aggregate fraction of transferred CD4^+^ and CD8^+^ Teffs that underwent cell division on days 3 and 5. Horizontal bars represent the mean. Mann-Whitney tests used. *n* = 4–7 mice per group from at least 3 independent experiments in **C**–**F**. *P < 0.05.

Although suggested in previous reports (4–6), it remains unclear whether Tregs can exert suppressive function within inflamed transplanted tissues. To directly address this, we used an approach that replicates either early rejection by Teffs or Treg suppression of rejection by Teffs, while restricting the immune responses to transplanted tissues. Allogenic islets were transplanted in diabetic mice lacking all SLOs, i.e., splenectomized lymphotoxin β receptor KO (LTβR^–/–^). Despite being lymphoreplete, these mice are unable to reject solid allografts unless exogenous Teffs or memory T cells are transferred (15, 16). To prompt rejection or suppression of rejection, the mice were adoptively transferred with either 2 × 10^6^ to 3 × 10^6^ Teffs (containing both CD4^+^ and CD8^+^ T cells) alone or with 2 × 10^6^ to 3 × 10^6^ Tregs (Figure 1A). Teffs were induced through immunization with donor splenocytes shortly (7 days) before adoptive transfer, whereas Tregs were harvested from donor antigen-immunized Foxp3 reporter mice after a rest period of greater than 30 days following immunization. As expected (15, 16), splenectomized LTβR^–/–^ recipients without cell transfer failed to reject islet allografts, and the transfer of Teffs led to acute rejection of islet allografts in all recipients. In contrast, the addition of Tregs protected from graft rejection by Teffs, delaying rejection initially and providing long-term protection from rejection in approximately 65% of recipients (Figure 1B). Interestingly, transferring Tregs from naive mice along with Teffs similarly protected from graft rejection (compared with Tregs from donor antigen-immunized mice), and this occurred without prior priming of Tregs in SLOs (Figure 1B). Comparable results were obtained in another mouse strain lacking SLOs, i.e., *aly/aly* mice (Supplemental Figure 1, A and B; supplemental material available online with this article; https://doi.org/10.1172/jci.insight.160579DS1). These results demonstrate that Treg suppression of Teffs can occur in the absence of prior priming in SLOs.

### Tregs suppress Teffs within the graft directly.

We then confirmed that immune responses are restrained to transplanted tissues in our model. Using the same approach as above, but transferring CFSE-labeled congenic T cells, we found that Teffs proliferated exclusively within islet allografts (Figure 1C; day 3). During rejection, both CD4^+^ and CD8^+^ Teffs underwent acute proliferation and extensively accumulated within islet allografts over time (days 3–7; Figure 1, E and F). Tregs demonstrated minimal proliferation within allografts compared with Teffs (Figure 1C) and showed more reduced accumulation rates within allografts over time than Teffs (Figure 1D) but retained Foxp3 expression (>85%; not shown). Interestingly, despite Treg protection from rejection (Figure 1B), the cotransfer of Tregs with Teffs had no effect on Teff numbers or the proliferation within allografts compared with Teffs transferred alone (Figure 1, D–F). In turn, this led to a decrease in Treg/Teff ratios over time (from 12% on day 3 to less than 5% on days 5–7; Figure 1D). Taken together, these data demonstrate that our model restricts immune responses to inflamed transplanted tissues and that both Teffs and Tregs undergo expansion at that site. While Tregs protect from rejection within transplanted tissues, this occurs independently of Teff priming and proliferation at that site. This implies that Tregs regulate Teffs within the grafted tissue by modulating Teff cytotoxic function.

### Teffs and Tregs are predominantly found in CD11c^+^ APC-rich areas surrounding transplanted islets.

**Figure 2:**
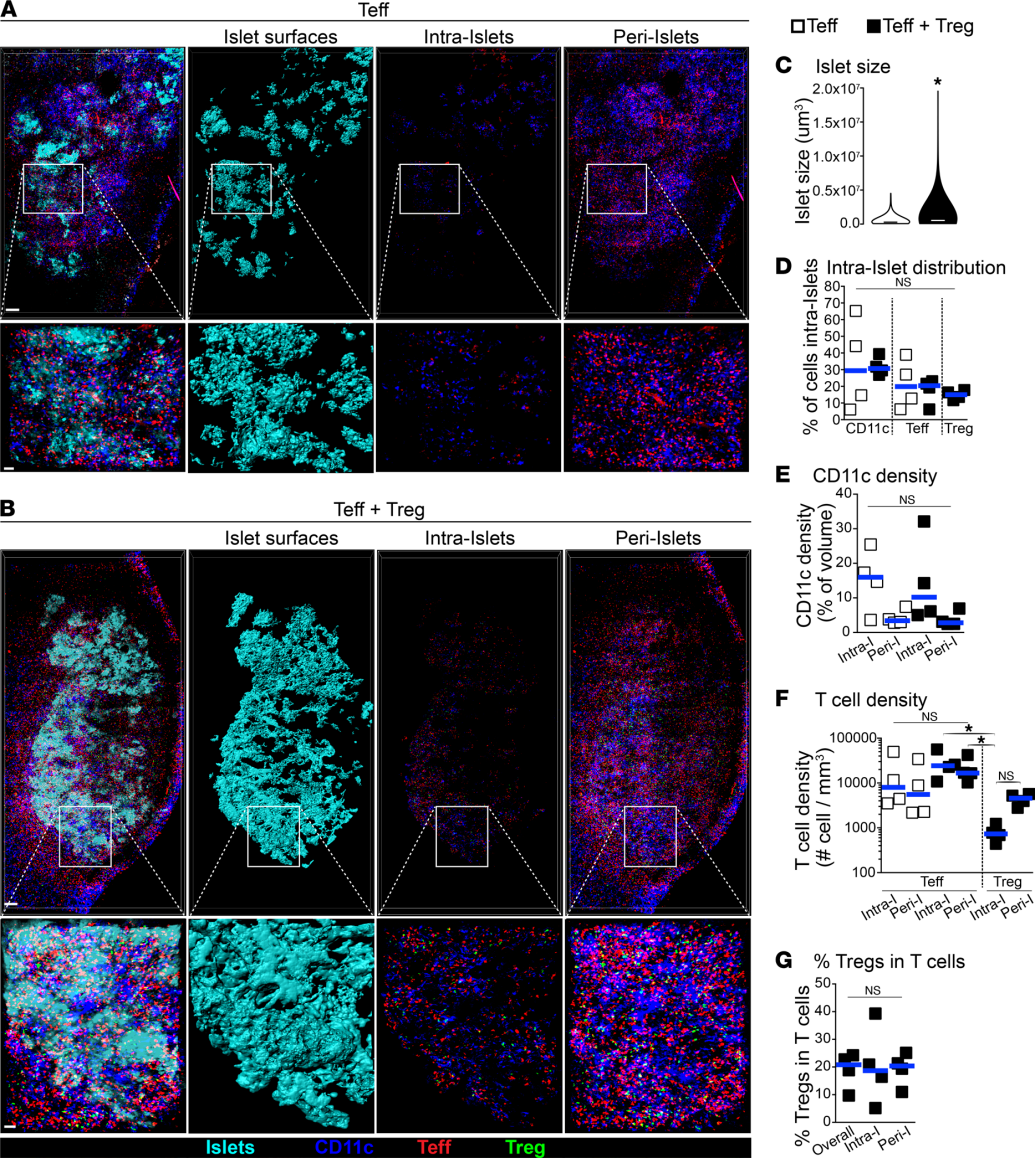
Both Teffs and Tregs are found in APC-rich areas surrounding transplanted islets. (**A** and **B**) Representative 3D-rendered IVM-stitched images of islet allografts and immune cells in mice lacking SLOs (as in Figure 1A) that received Teffs alone or Teffs + Tregs 4 days after cell transfer. Islet surfaces were generated on fluorescently labeled transplanted islets (second column), which distinguishes between intra-islet and peri-islet cellular infiltrates (third and fourth columns, respectively). White squares demonstrate magnified areas shown at the bottom of each panel. Scale bar: 200 μm (top) and 50 μm (bottom). (**C**) Tregs rapidly protected transplanted islets from rejection by Teffs. Violin plot of individual islet size on day 4 measured from images as in **A** and **B**. From measurements of 770–860 individual islets per group (4 mice per group). Mann-Whitney test used. Horizontal bars show median. (**D**) Fraction of cells within each subset that infiltrated within islets (intra-islet) from images, as in **A** and **B**. (**E** and **F**) Intra-islet and peri-islet density of CD11c^+^ cells and T cells from images, as in **A** and **B**. (**G**) Fraction of Tregs in the total T cell infiltrate from images, as in **B**. Each square in **D**–**G** represents data from 1 mouse, and horizontal bars show median. Kruskal-Wallis with Dunn’s multiple comparison tests used in **D**–**G**. *P < 0.05.

We used IVM to further investigate the mechanisms by which Tregs suppress within allografts. The approach described above was modified so that Teffs, Tregs, CD11c^+^ APCs, blood vessel lumens, and transplanted islets could be imaged simultaneously, using a combination of fluorescent mice and dyes (detailed in Methods section). In addition, Treg suppression of Teff was optimized (i.e., 100% protection from rejection) by transferring freshly activated Tregs (day 7) against donor antigens instead of rested Tregs (Supplemental Figure 1C). Given that insulitis (i.e., intra-islet infiltration) is a hallmark of islet destruction by Teffs in islet transplantation and in diabetes (17), we first analyzed large high-resolution 3D intravital images of entire islet grafts on day 4 following cell transfer. When Teffs were transferred alone and rejection was ongoing, only around 20% of Teffs were found infiltrating islets (intra-islet), whereas the large majority (~80%) of Teffs were found surrounding the transplanted islets (peri-islet; Figure 2, A and D). Peri-islet infiltration by Teffs was especially concentrated in areas rich in CD11c^+^ APCs (Figure 2A). Despite this, Teff infiltration density (i.e., the number of Teffs per mm^3^) in peri-islets versus intra-islets was not significantly different (Figure 2F), indicating that there was no preferential accumulation of Teffs among these areas. In a similar fashion to Teffs, CD11c^+^ APCs distributed predominantly surrounding transplanted islets, but without preferential peri-islet or intra-islet accumulation (Figure 2, A, D, and E). The addition of Tregs to Teffs protected transplanted islets from destruction by Teffs (Figure 2, B and C). The large majority of Tregs were found in peri-islet areas, where both Teffs and CD11c^+^ APCs were also present (Figure 2, B, D, and F). The presence of Tregs did not affect Teff or CD11c^+^ APC accumulation in either peri- or intra-islets (Figure 2, E and F), and the fraction of Tregs within transferred T cells were approximately 20% in all areas (Figure 2G; as similarly observed by flow cytometry in Figure 1E).

### Teffs are highly motile during active rejection, making brief and infrequent contacts with CD11c^+^ APCs and islets.

**Figure 3:**
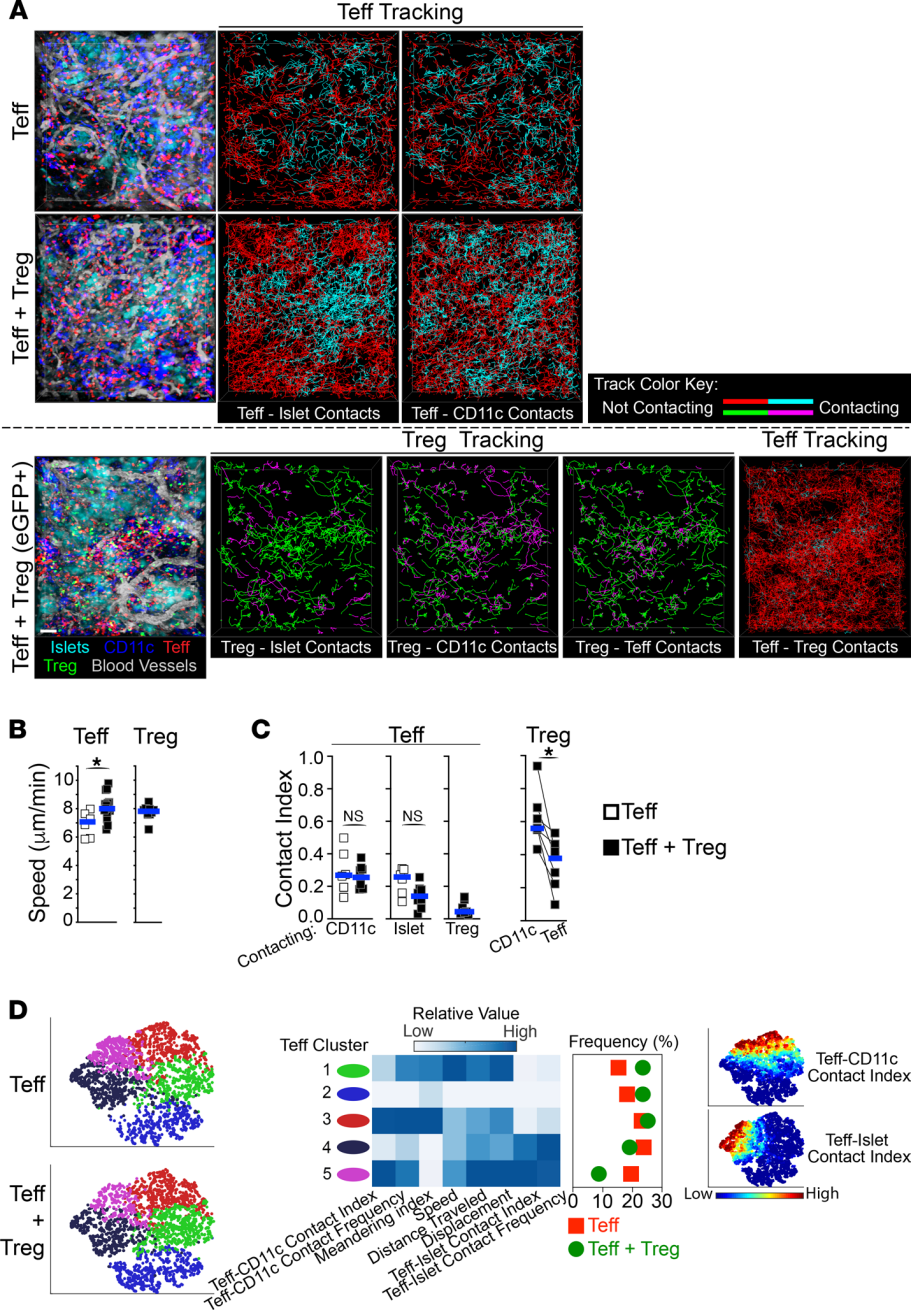
Tregs spend most of their time contacting CD11c^+^ APCs within islet allografts. Time-lapse IVM analysis of Teffs, Tregs, APCs, islets, and blood vessel lumens within transplanted islets 4 days after cell transfer of Teffs alone versus Teffs + Tregs (as in Figure 1A). (**A**) Representative still images (left) and tracking of individual Teffs and Tregs (lines in right panels). Tracking lines color changes to cyan (for Teffs) or to magenta (for Tregs) when in contact with another cell as indicated in each panel. IVM of Teffs + Tregs was performed in setups where Tregs were nonvisible (middle panels) or visible (EGFP^+^; bottom panels) to allow automated quantification of Teff contacts, as detailed in the Methods section. (**B**) Tregs increase Teff velocity. Teff and Treg velocity from movies in **A**. (**C**) Teffs spend little time in contact with Tregs, while Tregs spend the majority of their time in contact with CD11c**^+^** APCs. Teffs and Tregs contact indexes (i.e., fraction of time spent in contact overall) with CD11c**^+^**, islet, Treg, and Teff cells from movies in **A**. Each square represents mean value from 1 movie, and horizontal bars show median. Mann-Whitney tests used in **B** and **C**. (**D**) Tregs specifically inhibit a subpopulation of Teffs that contacted both CD11c^+^ and islet cells. ViSNE multiparameter clustering of all Teff tracks from movies in **A** (left), relative value of individual parameters used in viSNE within each Teff cluster, and frequency distribution of Teff clusters (middle). Right panel depicts Teff contact index value distribution in viSNE plots. *n* = 3–4 mice per group using 2 or more movies for each mouse. For each movie, an average of 966 Teffs and 342 Tregs were analyzed. Each square in **B** and **C** represents the mean value from 1 movie. Horizontal bars show median. *P < 0.05.

Time-lapse IVM acquisitions were used to assess graft-infiltrating T cell dynamics and their interactions with one another, with CD11c^+^ APCs, and with transplanted islets (on day 4 following cell transfer). As shown in Supplemental Video 1 and Figure 3A, actively rejecting Teffs were highly motile and surveyed the entire graft area, while CD11c^+^ APCs were sessile. Also, occasional dividing Teffs were observed (Supplemental Video 1). Teff contacts with either CD11c^+^ APCs or islets were relatively brief and infrequent (~2 minutes each at ~8 contacts/h; Supplemental Figure 2A). Only a very small fraction (<2%) of Teffs had prolonged interactions (>15 minutes) with either CD11c^+^ APCs or islets (not shown). Consequently, Teffs spent approximately 25% of their time in contact with either CD11c^+^ APCs or with transplanted islets; the contact index is the fraction of time spent in contact for each individual cell (Figure 3C). Of the small fraction of Teffs that had prolonged contact with islets, this occurred while Teffs crawled on the outer surface of the islet tissue (Supplemental Video 2). Occasionally, some Teffs migrated in and out of transplanted islets, demonstrating a potentially previously unappreciated plasticity of Teff dynamics during active rejection (Supplemental Video 2). Thus, Teffs attack transplanted islets from their outer edge, as similarly observed during immune attacks of pancreatic islets in type 1 diabetes (18, 19)

### Tregs are also highly motile and spend most of their time contacting CD11c^+^ APCs.

Next, we analyzed the dynamics of Tregs within allografts when transferred with Teffs. Graft-infiltrating Tregs were as motile as Teffs, and we also surveyed a large fraction of the graft area (Figure 3, A and B, and Supplemental Video 3) where they made contacts with both CD11c^+^ APCs and Teffs. These contacts were brief (~2 minutes each) but frequent (12–16 contacts/h; Supplemental Figure 2A). Nevertheless, Treg interactions with CD11c^+^ APCs were significantly longer and more frequent than those with Teffs. Thus, despite being quite motile and adjacent to both CD11c^+^ APCs and Teffs, Tregs spend most of their time (56%) interacting with CD11c^+^ APCs than with Teffs (37%; Figure 3C). In addition, Treg-APC interactions were considerably elevated compared with Teff-APC interactions, doubled both in their frequency (16 vs. 8 contacts/h; Supplemental Figure 2A) and in their contact index (56% vs. 25%; Figure 3C), demonstrating distinct behaviors between Tregs and Teffs. These data highlight a possible role for Treg-APC contacts in Treg suppressive function within allografts.

### Tregs suppress a subset of Teffs that preferentially interact with both APCs and islets.

We then assessed how the presence of Tregs affected the dynamics of Teffs within allografts. Only a small fraction of Teffs made direct contact with Tregs (less than 20%; not shown) and, on average, Teffs only spent around 4% of their time in contact with Tregs (Figure 3, A and C). Despite this, the presence of Tregs within allografts led to an increase in Teff speed (Figure 3B). However, Tregs did not significantly affect Teff contacts with CD11c^+^ APCs or with islets, compared with Teffs alone (Figure 3, A and C, and Supplemental Figure 2A). However, we reasoned that Tregs may specifically affect subsets of Teffs, which would not be reflected in population-wide analyses. To address this, Teffs from these IVM data sets were subdivided using unassisted clustering and multidimensional t-distributed stochastic neighbor embedding (t-SNE) plots (viSNE; ref. 20), generated using the IVM-derived parameters listed in Figure 3D. Comparing Teff alone versus Teff + Treg transfers, we observed a distribution shift in specific Teff subsets that mainly clustered according to their levels of Teff-APC and Teff-islet contacts. Specifically, in the presence of Tregs compared with Teffs alone, we observed a 30% frequency increase in Teffs that made minimal contacts with both APC and islets (Figure 3D, clusters 1–2, green and blue). This was paralleled by a reciprocal 35% frequency decrease in Teffs that made substantial contacts with both APCs and islets (Figure 3D, clusters 4–5, black and magenta). These data further highlight the heterogeneity of Teff behavior within allografts during rejection and demonstrate that Tregs prevent rejection by specifically suppressing a subset of Teffs that preferentially contact both APCs and the target tissue.

### Tregs preferentially contact CD11c^+^ APCs that are substantially and simultaneously interacting with Teffs.

**Figure 4:**
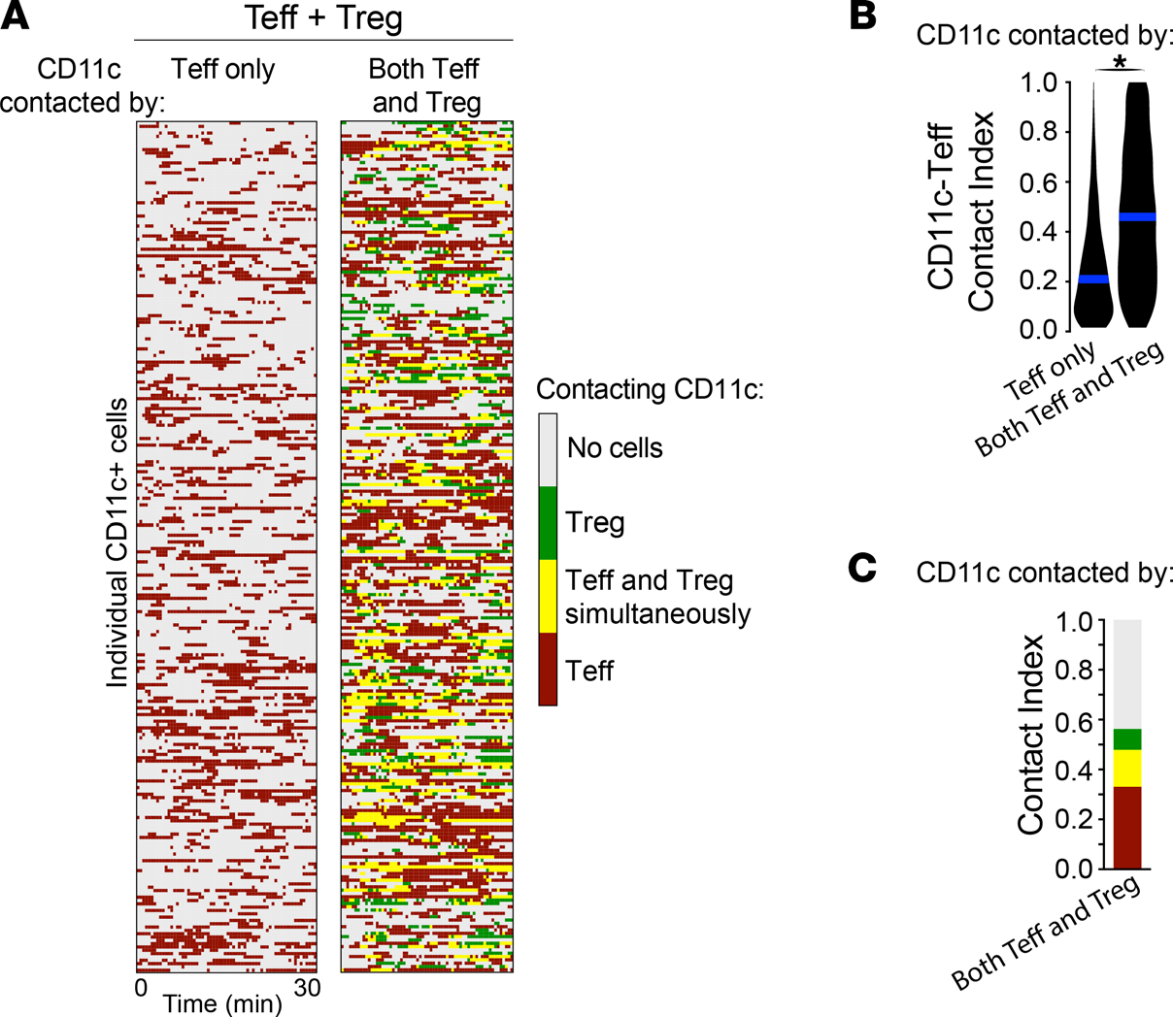
Tregs preferentially contact CD11c^+^ APCs that are substantially and simultaneously interacting with Teffs. (**A**) Heatmap of individual CD11c^+^ APCs and their contacts over time separated according to whether they were contacted by Teffs only (left column) or by both Teffs and Tregs (right column; from representative Teff + Treg movie in Figure 3A). (**B** and **C**) Overall mean CD11c-Teff contact indexes and color-coded overall mean contact indexes of CD11c^+^ APCs being contacted by Teffs only or by both Teffs and Tregs (from Teff + Treg movies in Figure 3A). Mann-Whitney test used. *n* = 3–4 mice per group using 2 or more movies for each mouse. For each movie, an average of 321 CD11c^+^ APCs were analyzed. *P < 0.05.

Given that Tregs accumulated in areas rich in both APCs and Teffs, we wondered whether Tregs contacted CD11c^+^ APCs that were simultaneously interacting with Teffs. To address this, we analyzed Teff and Treg contacts made to individual CD11c^+^ APCs in our IVM data sets where both Teffs and Tregs were transferred. We compared the fraction of time each individual CD11c^+^ APC spent in contact with Teffs, distinguishing whether APCs were contacted by Teffs only, or by both Teffs and Tregs (Figure 4). We found that approximately 45% of CD11c^+^ APCs were contacted by Teffs only, approximately 40% by both Teffs and Tregs, and approximately 1% by Tregs only (not shown). Interestingly, CD11c^+^ APCs that were contacted by both Teffs and Tregs spent more than twice the amount of time in contact with Teffs than CD11c^+^ APCs that were contacted by Teffs only (46% vs. 21%; Figure 4B). Remarkably, the large majority of CD11c^+^ APC-Treg interactions (64%) occurred while the same APC was simultaneously being contacted by Teffs (Figure 4C, yellow fraction in relation to green fraction; and Supplemental Video 4). Taken together, these data demonstrate that there is specialization in Treg contacts, where Tregs are preferentially attracted toward CD11c^+^ APCs that are substantially interacting with Teffs.

### Tregs reduce expression of both MHC-II on APCs and IFN-γ in Teffs.

**Figure 5:**
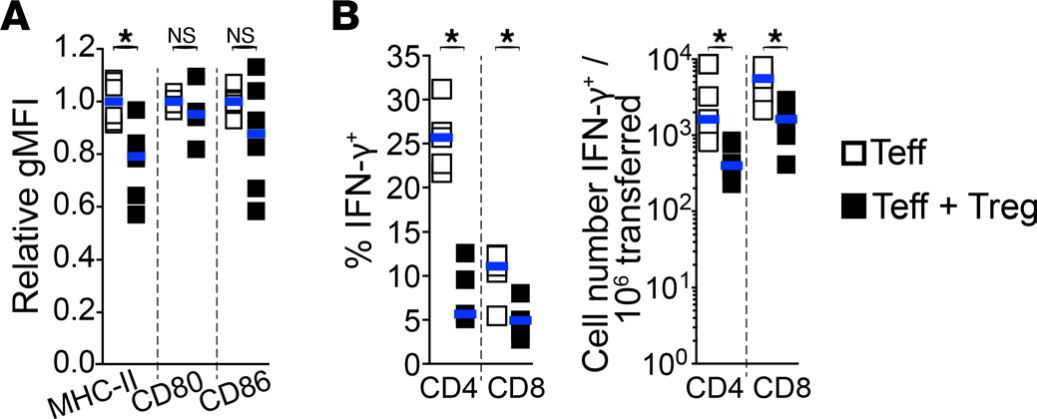
Tregs reduce expression of both MHC-II on APCs and IFN-γ in Teffs within allografts. Innate immune cells were characterized by flow cytometry from islet allografts of mice lacking SLOs (as described in Figure 1A), 7 days after cell transfer. (**A**) Tregs significantly reduce MHC-II, but not CD80 and CD86, expression levels on CD11c^+^MHC-II^+^ innate cells within allografts. Relative mean geometric MFI of MHC-II, CD80, and CD86 expression levels on live CD45^+^Lin^–^CD11c^+^MHC-II^+^ cells within islet allografts. *n* = 4–6 mice per group from 2–3 independent experiments. (**B**) Tregs reduce IFN-**γ** expression in Teffs within allografts. Percentage (left) and absolute cell numbers (right) of IFN-**γ**–expressing CD4^+^ and CD8^+^ Teffs by direct in vivo cytokine assessment. *n* = 5 per group from 2 independent experiments. Each square represents data from 1 mouse and horizontal bars show median. Mann-Whitney tests used. *P < 0.05.

Our data demonstrate that Tregs within allograft primarily interact with APCs, which correlates with decreased Teff-APC and Teff-islet interactions in a subset of Teffs. This suggests that Tregs may affect the function of both CD11c^+^ APCs and Teffs. To address this, we first examined whether Tregs affected APC subsets within islet allografts by flow cytometry on day 7 after cell transfer of Teffs or Teffs + Tregs into splenectomized LTβR^–/–^ recipients. We found that nearly all innate cells infiltrating islet allografts expressed CD11c but with variable levels of CD11b (Supplemental Figure 3A). These CD11c^+^ cells were then clustered into subsets using the parameters listed in Supplemental Figure 3B in viSNE. Comparing the frequency of the innate cell subsets between transfers of Teffs alone and Teffs + Tregs, we found that the presence of Tregs only had moderate effects (Supplemental Figure 3B). Nevertheless, in the presence of Tregs, we observed slight decreases in the frequency of innate cell clusters that expressed elevated MHC-II expression (Supplemental Figure 3B, clusters 5–7). In support of this, population-wide analysis of innate cells showed a significant reduction in MHC-II expression levels in the presence of Tregs. However, no significant changes were observed in the expression levels of the costimulatory molecules CD80 and CD86 (Figure 5A), as similarly reported by another study investigating the resulting effect of Treg therapy within pancreatic islets (21).

In addition, to assess whether Tregs affect Teff function within allografts, we evaluated IFN-γ expression in Teffs on day 7. We found that Tregs significantly reduced the fraction and absolute numbers of CD4^+^ and CD8^+^ Teffs expressing IFN-γ within allografts (Figure 5B). Taken together, Tregs potentially reduce the function of both CD11c^+^ APCs (through a reduction in antigen presentation) and Teffs within allografts.

### Intragraft Treg suppressive function relies on the ectonucleotidase CD73.

**Figure 6:**
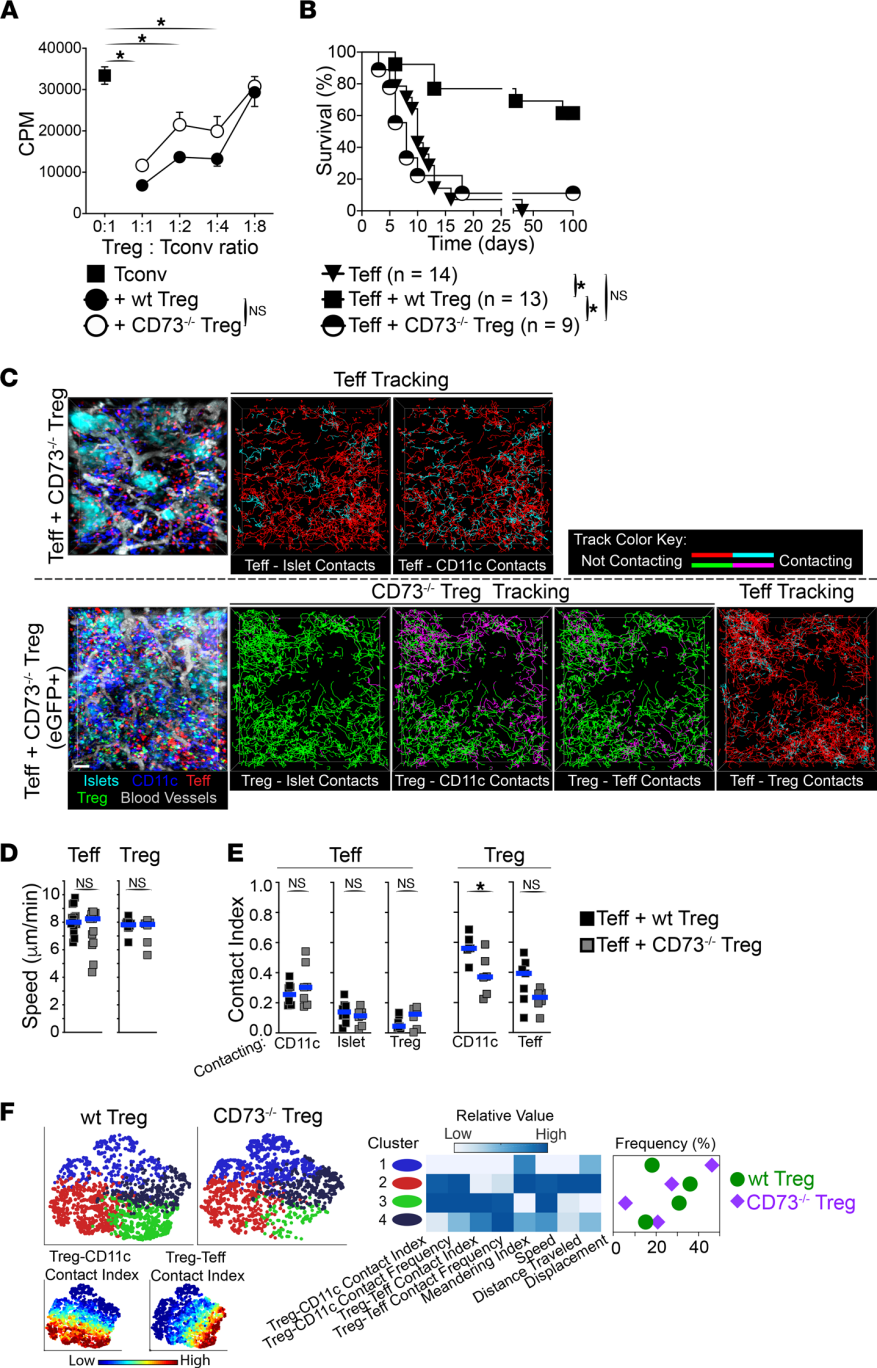
Treg suppressive function within allografts is dependent on the ectonucleotidase CD73. (**A**) WT and CD73^–/–^ Tregs are equally suppressive in vitro (results from 2 experiments). Kruskal-Wallis tests used. (**B**) CD73^–/–^ Tregs fail to protect from rejection within allografts. Graft survival in mice lacking SLOs bearing islet allografts (as in Figure 1A) received cells as indicated. Teff and Teff + WT Treg data are from Figure 1B. Log-rank Mantel-Cox tests used. (**C**) Time-lapse IVM of Teffs + CD73^–/–^ Tregs within transplanted islets 4 days after cell transfer. Representative stills (left) and tracking of individual Teffs and Tregs (lines in right panels). Tracking line color changes to cyan (for Teffs) or magenta (for Tregs) when in contact with other cells as indicated in each panel. IVM of Teffs + CD73^–/–^ Tregs was performed in setups where Tregs were nonvisible (top panel) or visible (GFP; bottom) to allow automated quantification of Teff contacts (see Methods). (**D**) Teff and Treg velocity from movies in **C** (Teffs + CD73^–/–^ Tregs) and Figure 3A (Teffs + WT Tregs). Teff + WT Treg data are from Figure 3B. (**E**) CD73^–/–^ Tregs contacted CD11c^+^ APCs significantly less than WT Tregs. Teff, WT Tregs, and CD73^–/–^ Treg contact indexes from movies as in **D** and from Figure 3C. Mann-Whitney tests used (**D** and **E**). (**F**) The behavior of WT Tregs and CD73^–/–^ Tregs within allografts further differs at subpopulation levels. ViSNE multiparameter clustering of Treg tracks as in **D** (left), relative value of IVM parameters used in viSNE within each Treg cluster (middle), and frequency distribution of Treg clusters (right). Bottom left: Treg contact index values in viSNE plots. *n* = 3 mice per group using ≥2 movies per mouse. For each movie, an average of 1120 Teffs and 500 Tregs were analyzed. Squares in **D** and **E** represent mean values from individual movies. Horizontal bars show median. *P < 0.05.

Tregs constitutively express the ectonucleotidase CD73, which generates antiinflammatory adenosine from AMP. Adenosine has been shown to inhibit both innate cells and T cells (22–25). Thus, we investigated the role of CD73 in Treg suppressor function within allografts. CD73^–/–^ Tregs were equally potent at suppressing T cell proliferation in vitro as WT Tregs (Figure 6A). However, unlike WT Tregs, CD73^–/–^ Tregs were unable to protect from islet allograft rejection by Teffs when transferred in mice lacking SLOs (Figure 6B), demonstrating that CD73 activity on Tregs is required for Treg suppression within allografts. We then used IVM to visualize the dynamics and interactions of Tregs with intact suppressor function (WT Treg) versus Tregs with impaired suppressor function (CD73^–/–^ Treg) within inflamed allografts using the same approach as in Figure 3 (day 4). CD73^–/–^ Tregs migrated to and accumulated within allografts in comparable fashion to WT Tregs and constituted 29% of the T cell infiltrate on average (not shown). In addition, and similarly to WT Tregs, CD73^–/–^ Tregs accumulated in peri-islet areas where Teffs and CD11c^+^ APCs were also present (Figure 6C). Despite CD73^–/–^ Tregs exhibiting similar motility to WT Tregs (Figure 6D), they made significantly fewer contacts with CD11c^+^ APCs compared with WT Tregs on a population-wide analysis (Figure 6, C and E, and Supplemental Figure 2B). In support of this, subset clustering of Treg tracks using viSNE demonstrated a 6-fold decrease in CD73^–/–^ Tregs making substantial contacts with both APCs and Teffs compared with WT Tregs (5% vs. 30%, respectively; Figure 6F, cluster 3, green). This was paralleled with a reciprocal increase in CD73^–/–^ Tregs that made minimal contacts with both APCs and Teffs (46% for CD73^–/–^ Tregs vs. 18% for WT Tregs; Figure 6F, cluster 1, blue). Interestingly, this altered behavior in CD73^–/–^ versus WT Tregs was specific to the inflamed allograft environment, as no significant differences in WT versus CD73^–/–^ Treg dynamics were observed in steady state spleen (Supplemental Figure 2C), and CD73^–/–^ mice are not autoimmune (not shown; ref. 26). We also examined the dynamics of Teff in the presence of CD73^–/–^ Tregs. Despite active rejection in these mice, we did not observe significant changes in Teff dynamics compared with Teffs cotransferred with WT Tregs (Figure 6, D and E, and Supplemental Figure 2B). Overall, these data demonstrate that CD73 expression is required for Treg suppressor function in inflamed environments, such as an allograft. This impaired suppressor function by CD73^–/–^ Tregs is associated with a significant reduction in Treg-APC contacts within allografts, suggesting that these contacts are central in Treg suppressor function. However, the underlying cause(s) of the altered behavior by CD73^–/–^ Tregs within allografts remains to be investigated.

## Discussion

Our knowledge of the mechanisms underlying Treg suppressor function within inflamed nonlymphoid tissues remains limited, especially in transplanted tissues. Given that Tregs adapt to their local environment and the wide variety of mechanisms underlying Treg immunosuppressor function in various tissues (7, 9, 10), uncovering the biology of Treg function in specific conditions has become increasingly important to identify potential therapeutic targets. Here, we demonstrate that Tregs do not require prior priming in SLOs for their migration and suppression within inflamed allografts. Indeed, circulating Tregs readily migrate to inflamed tissues and suppress both APCs and Teff cytotoxic functions. Our IVM data reveal that Tregs preferentially interact with APCs that are being significantly contacted by Teffs, demonstrating that particular APCs are being targeted by Tregs. In turn, Treg suppressor function prevents the generation of Teffs with elevated contacts with both APCs and their targets (i.e., transplanted islets). Mechanistically, Treg suppressor function within allografts relies on generating an antiinflammatory environment through the ectonucleotidase CD73. Our data also demonstrate a direct correlation between Treg suppressor function and elevated Treg-APC contacts, as CD73^–/–^ Tregs — which are unable to suppress Teff within inflamed allografts — show a drastic reduction in contacts with APCs compared with WT Tregs.

A previous IVM publication of transplanted islets placed in the anterior chamber of the eye reported prolonged interactions between Tregs and Teffs (27); however, this may be caused by the specific microenvironment at that location. In contrast, our report of preferential Treg-APC contact data parallels previous observations in SLOs during priming (28–30) and in tumors (31, 32). Biologically, many reasons may explain the requirements and specialization of these Treg-APC contacts. First, APCs provide the required TCR signals for Treg homeostasis, activation, and suppressor function (33–36). Thus, Treg-APC interactions in inflamed tissues may be required to provide crucial TCR signals for Treg suppressor function, as previously demonstrated in tumors (31). Second, distinctively functional APCs may secrete T cell-attracting chemokines, which would converge both Tregs and Teffs to the same APCs. Indeed, recent studies revealed that chemokine receptor signaling on T cells (CCR5, CXCR3, or CXCR6) promote their interactions with APCs either in SLOs or non-SLOs (37–39). Similarly, CCR4 or CCR8 signaling in Tregs was shown to foster Treg-APC interactions (40, 41). Finally, Treg-APC interactions may be the result of active suppression of APC function. APCs are targets of Treg suppression, and targeted APCs demonstrate impaired antigenic presentation and reduced capacity to activate conventional T cells through active removal of MHC-II molecules (42), generating soluble tolerogenic factors (43), and/or CTLA-4–mediated suppression (44). Taken together, APCs potentially both promote Treg suppressor function and are the target of Treg suppression. The nature and requirements of these Treg-APC contacts in Treg suppressor function are currently under investigation in our laboratory.

ATP is rapidly released in the extracellular space upon tissue injury, which further promotes inflammation through P1 purinergic receptor signaling in immune and parenchymal cells (45). The proinflammatory functions of ATP can be reversed by hydrolysis of ATP into adenosine through an enzymatic cascade mediated by CD39 and CD73 extracellular enzymes (46). Adenosine receptor signaling promotes an antiinflammatory environment through the inhibition of both innate and adaptive immune responses (22–25, 47). Both CD39 and CD73 are constitutively expressed on Tregs in mice, and CD39^+^ human Tregs demonstrate enhanced suppressor capacity (48, 49). While a previous report attributed a role for adenosine generation in Treg suppressor function in transplantation (25), our data further demonstrate that this Treg suppression mechanism is primarily used within inflamed tissues. In turn, a better understanding of the mechanisms underlying Treg suppressor function within various inflamed tissues will help identify the therapeutic targets that promote such function.

## Methods

### Animals.

Sex-matched 6- to 10-week-old BALB/c (H-2d; National Cancer Institute/NIH or The Jackson Laboratory), C57BL/6 (B6; H-2b; National Cancer Institute/NIH or The Jackson Laboratory), B6.SJL-*Ptprc^a^Pepc^b^*/BoyJ (B6.CD45.1; H-2b; National Cancer Institute/NIH or The Jackson Laboratory), B6.Cg-Tg(Itgax-Venus)1Mnz/J (B6.CD11c-YFP; The Jackson Laboratory), C57BL/6-Foxp3tm1Flv/J (B6.Foxp3-RFP; The Jackson Laboratory), B6.129S1-Nt5e^tm1Lft^/J (B6.CD73^–/–^; The Jackson Laboratory), B6.129(ICR)-Tg(CAG-ECFP)CK6Nagy/J (B6.CFP; The Jackson Laboratory), C57BL/6-Tg(CAG-EGFP)1Osb/J (B6.eGFP; The Jackson Laboratory), and B6.PL-Thy1^a^/CyJ (B6.CD90.1; The Jackson Laboratory) mice were used. B6.Cg-Ltβr^tm1Mmat^/Rbrc (B6.LTβR^–/–^) were a gift from Mitsuru Matsumoto (The University of Tokushima, Tokushima, Japan) (50). B6.Foxp3-RFP mice were crossed with B6.CD90.1 in our facility. B6.CD73^–/–^ mice were crossed with B6.Foxp3-RFP.CD90.1 in our facility. In addition, B6.Foxp3-RFP.CD90.1 and B6.Foxp3-RFP.CD90.CD73^–/–^ mice were further crossed with B6.eGFP in our facility. Mice were housed with food and water ad libitum.

### Islet isolation and transplantation.

Islets from BALB/c donors were digested with Collagenase V or P (MilliporeSigma), purified by filtration through a 100 μm nylon cell strainer (BD Biosciences), cultured overnight in RMPI-1640 (containing 10% FBS, penicillin/streptomycin, HEPES, Glutamax, and 2-Mercaptoethanol; Thermo Fisher Scientific), hand-picked under a stereomicroscope, and placed (350–400 islets/recipient) under the left renal capsule of streptozocin-induced (190 mg/kg i.p.; MilliporeSigma) diabetic recipients as described (51). For intravital imaging, isolated islets were stained with CellTracker Orange CMRA (10 μM for 1 hour in RPMI-1640 media; Thermo Fisher Scientific) prior to transplantation. Graft survival was monitored through blood glucose measurements with levels greater than 350 mg/dL over 2 consecutive measurements indicating rejection. Unilateral nephrectomy to remove the transplanted islets was performed to confirm graft function in long-term survivors (>100 days).

### Abs, flow cytometry, and viSNE analysis.

Fluorochrome-conjugated mAbs against CD4 (clone RM4-5), CD19 (clone 6D5 or 1D3), Foxp3 (clone FJK-16s), CD45 (clone 30-F11), CD45.1 (clone A20), CD45.2 (clone 104), CD90.1 (clone OX-7), CD90.2 (clone 30-H12 or 53-2.1), B220 (clone RA3-6B2), NK1.1 (clone PK136), CD11b (clone M1/70), CD11c (clone POD1, HL3, or N418), Ly6C (clone HK1.4), Ly6G (clone 1A8), MHC-II (I-A/I-E; clone M5114.15.2), CD80 (clone 16-10A1), CD86 (clone GL-1), F4/80 (clone BM8 or T45-2342), CD16/32 (clone 93), CD8 (clone 53-6.7), IFN-γ (clone XMG1.2), and Ig control Abs were from BD Biosciences, Thermo Fisher Scientific, Tonbo Biosciences, or BioLegend. Dead cells were labeled using Fixable Dead Cell Stain (Thermo Fisher Scientific). Negative controls used appropriate Ig fluorochrome conjugates. Flow acquisition was performed on LSRII Fortessa or LSRII analyzers (BD Biosciences). A total of 1 × 10^6^ to 5 × 10^6^ events were acquired per sample. Quantitative cell numbers were calculated according to total live cell counts recovered from individual compartments. Tissues were dissociated using 350 U/mL Collagenase D (Thermo Fisher Scientific) with 0.02 mg/mL DNase I (MilliporeSigma) in RPMI-1640 medium containing 5% FBS at 37°C for 30–60 minutes under constant agitation. Data were analyzed using FlowJo software (BD Biosciences), and cell doublets and dead cells were excluded from the analysis. For innate cell subset analysis, Lin^+^ (i.e., CD4^+^, CD8^+^, NK1.1^+^, and CD19^+^) and Ly6G^+^ were excluded. For viSNE analysis, flow cytometry data from live Lin^–^Ly6G^–^CD45^+^CD11c^+^ singlets were hyperbolic arcsine transformed by a cofactor of 150 before t-SNE unsupervised cluster analysis using viSNE in CYT software as described (20). For IFN-γ staining, mice received 250 μg of Brefeldin A (MilliporeSigma) i.p. 4 hours prior to flow cytometry staining as described (52). Digestion of transplanted islets was done with the addition of 10 μg/mL of Brefeldin A throughout.

### Adoptive cell transfer.

Both CD4^+^ and CD8^+^CD44^hi^ Teffs were sort-purified from spleens of B6, B6.CD45.1, or B6.CFP mice immunized with donor splenocytes 5 days prior. Tregs were sort-purified from spleens of either naive B6.Foxp3-RFP.CD90.1 mice or B6.Foxp3-RFP.CD90.1, B6.Foxp3-RFP.CD90.1.eGFP, B6.Foxp3-RFP.CD90.1.CD73^–/–^, or B6.Foxp3-RFP.CD90.1.CD73^–/–^ EGFP mice immunized with donor splenocytes 7–30 days prior to cell transfer. A total of 2 × 10^6^ to 3 × 10^6^ Tregs and/or Teffs were transferred to each recipient 5 days after islet transplantation. In experiments reported in Figures 1 and 5 and Supplemental Figures 1 and 3, Tregs were harvested more than 30 days after immunization with donor splenocytes before cell transfer. In all IVM experiments, Tregs were harvested 7 days after immunization with donor splenocytes before cell transfer. In some experiments, Teffs and Tregs were stained with CFSE [5-(and-6)-carboxyfluorescein diacetate, succinimidyl ester; 2.0–2.5 μM; Thermo Fisher Scientific] prior to adoptive transfer

### Two-photon IVM.

B6.LTβR^–/–^ mice received total body irradiation (11 Gy) using a *γ* source and were reconstituted with 5 × 10^6^ to 10 × 10^6^ bone marrow cells from B6.CD11c-YFP mice at least 8 weeks before use as islet allograft recipients. Mice were anesthetized using isoflurane and the kidneys containing the transplanted islets or spleens were surgically exposed and mobilized using custom-made devices 4 days after cell transfer as we previously described (53, 54). Blood perfusion, mouse rehydration, and physiological temperature of 37°C were preserved and maintained throughout the imaging. We then performed 2-photon laser-scanning imaging using an upright Nikon A1 MP microscope with a Chameleon Ti:Sapphire femtosecond-pulsed laser (Coherent) tuned to 860 nm. Fluorescence emission was captured by 4 nondescanned GaAsP detectors coupled to the following bandpass emission filters: 480/20, 525/50, 600/60, and 705/55 nm. Time-lapse images were acquired using NIS-Elements software (Nikon). Stacks of 18 optical sections were acquired every 30 seconds for 30 minutes to provide image volumes of 50 μm (25–55 μm deep) in depth and around 497 μm in width and height at a resolution of 0.994 μm/pixel using a water-immersion 25× objective (NA = 1.1; Nikon).

### Two-photon IVM data analysis.

Images were spectrally unmixed using NIS-Elements. Image rendering, motion artifact correction, surface generation, and individual cell tracking of adoptively transferred cells, CD11c^+^ cells, and transplanted islets from time sequence of image stacks were performed using Imaris software (Bitplane). Cell tracks less than 2 minutes were excluded from analysis. For each data set, randomly picked tracks were visually inspected to ensure accuracy. Transplanted islet size was evaluated using a surface seed splitting size of 75 μm. For contact measurements, voxels inside surfaces were masked with a specific fixed value that varied among individual cell type and displayed in individual channels. Surfaces overlapped during contact and individual cell tracks were then analyzed for masked fixed values from other cell types over time. From this, contact time and frequency were generated. Contact index reports the fraction of time an individual cell is in contact with a specific cell type over the length of that entire track. Due to spectral unmixing incompatibilities, IVM of Teffs + Tregs had to be performed in 2 separate data sets: one where Tregs were nonfluorescent and another where Tregs were fluorescently labeled (EGFP-Treg). In data sets where nonfluorescent Tregs were used, Teff tracking parameters and Teff contacts were extracted. In data sets where fluorescent Tregs were used, Treg tracking parameters and Treg contacts were extracted. ViSNE was used for multiparameter IVM tracking analysis. To do so, individual track parameter values were linearly transformed using 1 as the maximum so that each parameter has equal weight in t-SNE calculations. Unsupervised cluster analysis and frequency was done using viSNE in CYT. IVM movies were edited using Premier Pro or Photoshop (Adobe).

### In vitro suppression assay.

Sorted WT or CD73^–/–^ Tregs were added to 50,000 T cells at various ratios in the presence of anti-CD3 and anti-CD28 Abs in round bottom 96-well plates. After 2 days in culture at 37°C, the wells were pulsed with ^3^H-thymidine and DNA-incorporated thymidine was measured on day 3 using a scintillation beta-counter.

### Online supplemental material.

Supplemental Figure 1 (complement to Figure 1) shows that Tregs can prevent allograft rejection by Teffs in a second SLO-deficient mouse model (splenectomized *aly/aly* recipient), and freshly activated Tregs provide complete protection from rejection. Supplemental Figure 2 (complement to Figure 3 and Figure 6) shows that Treg contacts with CD11c^+^ APCs are longer and more frequent than those with Teffs, and this is lost when Tregs lack CD73 expression. Supplemental Figure 3 (complement to Figure 5) shows that Tregs did not substantially affect the phenotype of innate immune cells infiltrating allografts. Supplemental Video 1 (complement to Figure 3) shows time-lapse IVM of islet allograft rejection by Teffs. Supplemental Video 2 (complement to Figure 3) shows the dynamics of Teffs making prolonged contacts with transplanted islets during rejection. Supplemental Video 3 (complement to Figure 3) shows time-lapse IVM of Treg suppression of islet allograft rejection. Supplemental Video 4 (complement to Figure 3) shows an example of Treg-APC continuous interactions while the same APC simultaneously interacts with Teffs.

### Statistics.

Nonparametric Mann-Whitney (comparing 2 groups), Kruskal-Wallis followed by Dunn’s multiple-comparison (comparing 3 or more groups), or log-rank Mantel-Cox (for survival curves) tests were used for statistical analyses using Prism (GraphPad). Differences were considered significant at *P* < 0.05. Bars show mean ± SEM or median as indicated.

### Study approval.

Studies were performed in compliance with NIH guidelines and approved by the Institutional Animal Care and Use Committee at the University of Pittsburgh.

## Author contributions

HD, AP, LB, and AW performed experiments. SCW provided intellectual contributions and edited the manuscript. GC designed and performed experiments, collected and analyzed data, and wrote the paper.

## Supplementary Material

Supplemental data

Supplemental video 1

Supplemental video 2

Supplemental video 3

Supplemental video 4

## Version 1 07/26/2022

In-Press Preview

## Version 2 08/22/2022

Electronic publication
