# Supplementary material for: Treg suppression of immunity within inflamed allogeneic grafts
Source: JCI Insight. 2022 Aug 22;7(16):e160579. doi: 10.1172/jci.insight.160579 (PMC9462475; doi:10.1172/jci.insight.160579)
Supplement: Supplemental data [file jciinsight-7-160579-s223.pdf]

SUPPLEMENTAL MATERIAL

Treg suppression of immunity within inflamed allogeneic grafts

Hehua Dai[1], Andressa Pena[1], Lynne Bauer[1], Amanda Williams[1], Simon C. Watkins[2] and Geoffrey Camirand[1]

[1] The Thomas E. Starzl Transplantation Institute, Departments of Surgery and Immunology, University of Pittsburgh School of Medicine, Pittsburgh, PA 15261

[2] Center for Biologic Imaging, Department of Cell Biology and Physiology, University of Pittsburgh School of Medicine, Pittsburgh, PA 15261

Supplemental Figure 1

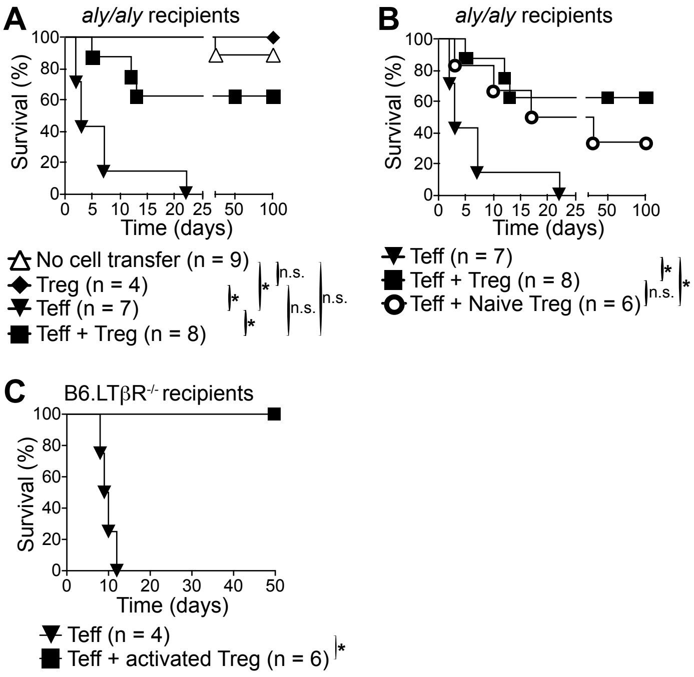

Supplemental Figure 1 (Complement to Fig. 1). Treg can prevent allograft rejection by Teff in a second SLO-deficient mouse model (splenectomized aly/aly recipients), and freshly activated Treg provide complete protection from rejection.

(A-B) Islet allograft survival in splenectomized aly/aly recipients that received cell transfers as indicated and as described in Fig. 1A. Treg were from mice previously exposed to donor antigens, while “naïve Treg” were from naïve mice. Log-rank (Mantel-Cox) test used.

(C) We tested whether freshly activated Treg would provide better protection from rejection in our model. Islet allograft survival in splenectomized LTβR-/- recipients that received 2-3x10^6 CD44Hi sorted Teff alone, or with 2-3x10^6 Treg from freshly immunized mice against donor antigens. Using freshly activated Treg provided protection from rejection in 100% of the recipients. This approach was then used for our IVM studies.

Supplemental Figure 2

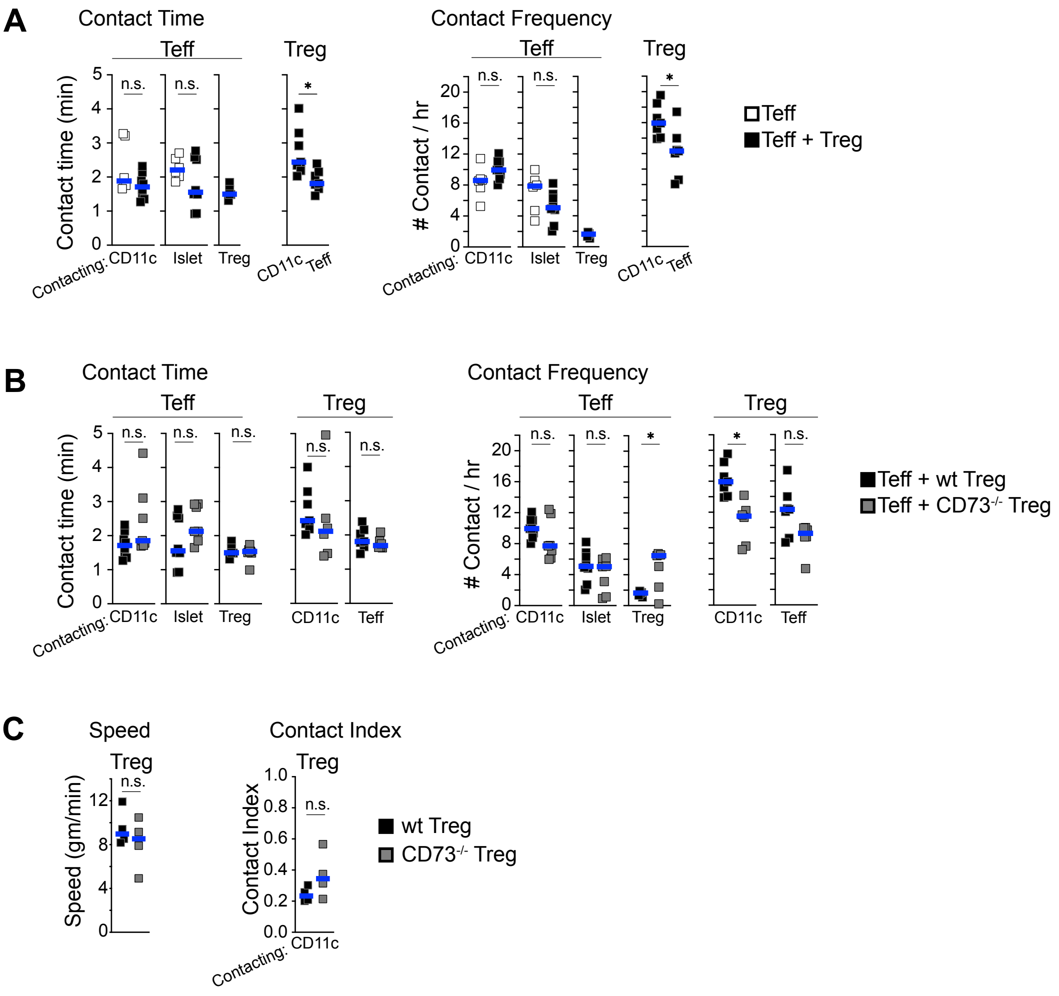

Supplemental Figure 2 (Complement to Fig. 3 and Fig. 6). Treg contacts with CD11c⁺ APCs are longer and more frequent than those with Teff, and this is lost when Treg lack CD73 expression.

(A) Treg contact CD11c⁺ APCs more than Teff. Teff and Treg contact time (left) and contact frequency (right) with CD11c+ APCs, islet, Treg and Teff cells from movies in Fig. 3A.

(B) $CD73^{-/-}$ Treg, which cannot suppress within allografts, demonstrate reduced interactions with $CD11c^{+}$ APCs compared to wt Treg. Teff and Treg contact time (left) and contact frequency (right) with $CD11c^{+}$ APCs, islet, Treg and Teff cells from movies in Fig. 6C. Each square represents mean value from one movie and horizontal bars show medians. N = 3-4 mice / group using 2 or more movies for each mouse (A-B).

(C) $CD73^{-/-}$ Treg exhibit similar dynamics than wt Treg in steady state. IVM of $CD73^{-/-}$ Treg and wt Treg in spleen of naïve B6.CD11c-YFP mice 2 days after cell transfer. Speed and Treg-$CD11c^{+}$ APC contact indexes of wt and $CD73^{-/-}$ Treg. Each square represents mean value from one movie and horizontal bars show medians. N = 2 mice / group, from 2 independent experiments. 2 movies / mouse were analyzed. Mann-Whitney tests used (A-C). *: $p < 0.05$. n.s.: not significant.

Supplemental Figure 3

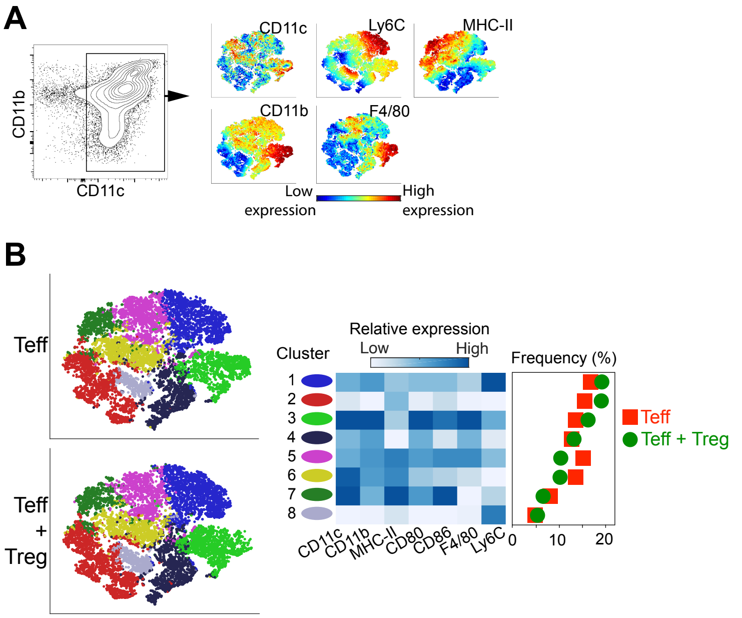

Supplemental Figure 3 (Complement to Fig. 5). Treg did not substantially affect the phenotype of innate immune cells infiltrating allografts. Innate immune cells were characterized by flow cytometry from islet allografts of mice lacking SLO (as described in Fig. 1A), 7 days after cell transfer.

(A) Nearly all innate immune cells within islet allografts expressed CD11c (Left panel; gated on live CD45+ Lin- NK1.1- Ly6G-). CD11c+ cells were clustered with ViSNE using the markers listed in B. tSNE plots and relative expression levels of selected markers shown (right).

(B) ViSNE cluster analysis demonstrates that Treg induce a slight decrease in frequency of clusters 5 and 6, which express MHC-II and some CD80 and CD86, and a correlate slight increase in frequency of clusters expressing low levels of MHC-II, CD80 and CD86. Left: representative ViSNE plots and clustering for each group. Right: Heat map of relative expression of various markers within individual clusters, and frequency of each cluster within the entire CD11c+ cell population. Representative of 2 independent experiments, n=2 mice / group / experiment.

SUPPLEMENTAL VIDEO LEGENDS

Supplemental Video 1 (Complement to Fig. 3). Time-lapse IVM of islet allograft rejection by Teff. Representative time-lapse IVM of transplanted allogenic islet rejection by Teff on day 4. Splenectomized B6.LTβR$^{-/-}$ mice reconstituted with bone marrow from B6.CD11c-YFP were transplanted with CTO-stained Balb/c islets under the kidney capsule and received Teff isolated from B6.CFP mice. Blood vessel lumens were identified using Evans blue. The first segment of the video demonstrates blood flow and blood vessels within the imaging volume. The second segment shows Teff dynamics (same video played twice). The third segment shows various perspectives of Teff tracks (red lines), CD11c^{+} APCs (blue surfaces), and transplanted islets (cyan surfaces) within the imaging volume.

Supplemental Video 2 (Complement to Fig. 3). Dynamics of Teff making prolonged contacts with transplanted islets during rejection. Representative time-lapse IVM of Teff crawling on the surface and through transplanted allogeneic islets during rejection (same approach as in Supplemental Video 1). The first segment of the video demonstrates Teff crawling on the surface of transplanted islets. The second and third video segments show examples of Teff crawling through transplanted islets. Teff tracking lines change color from red to cyan when Teff are contacting islets.

Supplemental Video 3 (Complement to Fig. 3). Time-lapse IVM of Treg suppression of islet allograft rejection. Representative time-lapse IVM of Treg suppression of transplanted allogenic islet rejection by Teff on day 4. Same approach as in Supplemental Video 1, except that the mice additionally received Treg from Foxp3-RFP.CD90.1.eGFP mice with Teff. The first segment of the video demonstrates blood flow and blood vessels within the imaging volume. The second segment shows Teff and Treg dynamics (same video played twice). The third segment shows

various perspectives of Teff and Treg tracks (red and green lines respectively), $CD11c^+$ APCs (blue surfaces), and transplanted islets (cyan surfaces) within the imaging volume.

Supplemental Video 4 (Complement to Fig. 4). Example of Treg-APC continuous interactions while the same APC simultaneously interacts with Teff. Zoomed-in representative time-lapse IVM of Treg-APC-Teff interactions using software-generated surfaces. Same approach as in Supplemental Video 3. Top panel showing Treg and APC only, and bottom panel showing Teff, Treg and APC from the same video.
